# Work environment adversity and non-communicable Disease risk among drivers working for application-based-cab-aggregators in an Indian metropolis: Assessment of work environment adversity and its association with ncd risk factors among application based cab aggregators in Bengaluru, India: a cross sectional study

**DOI:** 10.1186/s12889-024-18728-y

**Published:** 2024-06-14

**Authors:** Gautham Melur Sukumar, Mohana Balan Parivallal, Shalin Lily Giboy, Aditi Narendra Thakkar

**Affiliations:** 1 Centre for Public Health, Department of Epidemiology, National Institute of Mental Health and Neuro Sciences, Bengaluru, India; 2 Department of Epidemiology, National Institute of Mental Health and Neuro Sciences, Bengaluru, India

**Keywords:** Non communicable disease, Occupational risk, Work environment adversity, Cab drivers, Urban transport, Physical inactivity, Tobacco use, Unhealthy diet, Basic occupational health Services

## Abstract

**Background:**

Bengaluru, a metropolis in Southern India, is one of the largest markets for cab aggregator companies. Drivers working for these companies play a vital role in urban transportation but unlike other drivers, their work pattern is stressful, which could increase their proneness to NCD risk factors. Understanding associations between work environment adversity and NCD risk factors among these drivers will help to plan specific health promotion and NCD prevention programs including provision of basic occupational health services.

**Objectives:**

The study aims to test for an association between work environment adversity and selected Non-communicable Disease (NCD) risk factors among Application Cab Aggregator drivers in Bengaluru city and to estimate the prevalence of selected NCD risk factors among the ABCA drivers.

**Methodology:**

This cross-sectional study was conducted in Bengaluru city among 340 eligible and consenting ABCA drivers with at least one-year experience. Drivers were recruited through a multi-stage sampling procedure and time-period sampling, from transportation and leisure zones in the city. Data was collected through interviews using specifically developed semi-structured tools to assess work environment adversity and NCD risk factors. Prevalence of NCD risk factors is presented per 100 drivers with 95% confidence intervals. Multivariate Logistic regression analysis was conducted to quantify the strength of the association between work environment adversity categories and NCD risk factors. Ethical clearance was obtained from the NIMHANS Ethics Committee.

**Results:**

Nearly 97% of the 340 drivers reported having one or more NCD risk factors. Working more than 5 days a week, more than 7 + hours a day, staying away from family, and working night shifts were closely associated with higher risk for NCD risk factors among ABCA drivers. Drivers with work environment adversity scores between 5 and 10 were associated with higher odds of Physical Inactivity (OR = 3.1), Unhealthy diets (OR = 1.62), and Tobacco Use (OR = 3.06).

**Conclusion:**

The study highlights the association between work environment adversity and NCD risk factors and indicates a dire need for NCD prevention programs, basic occupational health services, and social security provisions for ABCA cab drivers.

**Supplementary Information:**

The online version contains supplementary material available at 10.1186/s12889-024-18728-y.

## Introduction

Urban population percent in India is expected to increase to 50% by the year 2050 from 31% in 2011 [1]. Nearly 42% of the urban population lives in one of the 53 million plus cities [2]. Urban health issues of mobility & road crashes, Non-Communicable Diseases (NCDs), infections, mental disorders, substance use, and violence are emerging as health priorities in the country. A rising prevalence of NCDs is observed and nearly one out of every four Indians are dying from NCDs prematurely (before 70 years) [3]. This epidemiological change in NCD burden is likely to be reflected in urban population and sub-population groups like the ‘workers’ [4]. Workers account for nearly 40% of the general population and nearly 92% are in the unorganized sector, with limited health and social security benefits [5]. Cab drivers constitute a key unorganized sector workforce in urban India who work for the mobility of people and the growth of the economy.

NCDs are attributed to four modifiable behavioural risk factors: excessive alcohol use, tobacco smoking, unhealthy diet and inadequate physical activity [6]. Often, adverse work environments propel the employees to adopt unhealthy lifestyles, making them vulnerable to non-communicable diseases. The association between working conditions and NCD risk factors is often discussed but seldom examined outside organized sector workplaces, especially among urban-transportation-mobility-related occupations. Understanding occupational risk for NCDs among cab drivers is vital to devise specific setting-based approaches to reduce NCDs.

Bengaluru city is home to nearly ten million people, spread across 2196 sq. km, connected by a 10,200 km road network, comprising nine zones, and 198 wards, and is a centre for a thriving IT, BT, Industrial, hospitality, and education sector. It is the fifth most populous urban agglomeration and the second fastest-growing major metropolis in India [7]. The population and economic activities place a huge urban transportation need resulting in the emergence and growth of Application Based Cab Aggregators (ABCA) from the year 2009, making Bengaluru one of the largest markets in India for cab aggregators like Ola and Uber. At the time of this study, an estimated 1.6 lakh ABCA cabs were reportedly plying on Bengaluru roads.

The working environment for app-based taxi drivers is distinct from that of regular taxi drivers; it is voluntary, with target-driven work patterns for trips and remuneration and no set work hours. Ultimately, they wind up working longer shifts while getting less or disrupted sleep. Numerous ABCA drivers live and work from cabs, which force them to rely on high-calorie, low-nutrient food served at unpredictable times. Many turn to substance use disorders, such as harmful use of alcohol and smoking tobacco, in order to cope up with the ongoing tensions at work. Absence of job security coupled with sustained exposure to urban traffic chaos and pollution, risk for road crashes, injuries, psychological stress of auto-loan repayment, staying away from family aggravate the situation [8]. These chronic stressors at work could increase the proneness of ABCA drivers to NCD risk factors and subsequent development of NCDs.

Evidence regarding the prevalence and association of key NCD risk factors and work environment adversities is limited for ABCA drivers. Understanding this relationship would aid in implementing Basic occupational health services for ABCA cab drivers. With no specific legislation governing the health and safety of these ABCA drivers, this information could help to catalyse advocacy to promulgate legislation for this population.

With the above context, this study aims to assess work environment adversity among ABCA drivers; estimate the prevalence of key NCD risk factors among ABCA drivers; and estimate the association between work environment adversity and NCD risk factors among ABCA drivers in Bengaluru city.

## Methodology

A cross-sectional study was conducted between March 2020 to September 2020 in Bengaluru City, India among drivers working for ABCA companies, with at least one year of experience and providing informed consent. ABCA drivers were recruited by multi-stage sampling. In the first stage (stratified sampling), the investigators mapped, listed, and created two strata-leisure and transportation zones, using secondary data sources. Around 80% of the sample was drawn from the transportation stratum and 20% from the leisure stratum. A transportation zone is operationally defined as an area/terminus that is specifically used as a transport transit point (namely airport terminal, bus terminal, railway station, and metro station). Leisure zones are areas popularly known for shopping, dining, entertainment, and recreational activities.

**Fig. 1:**
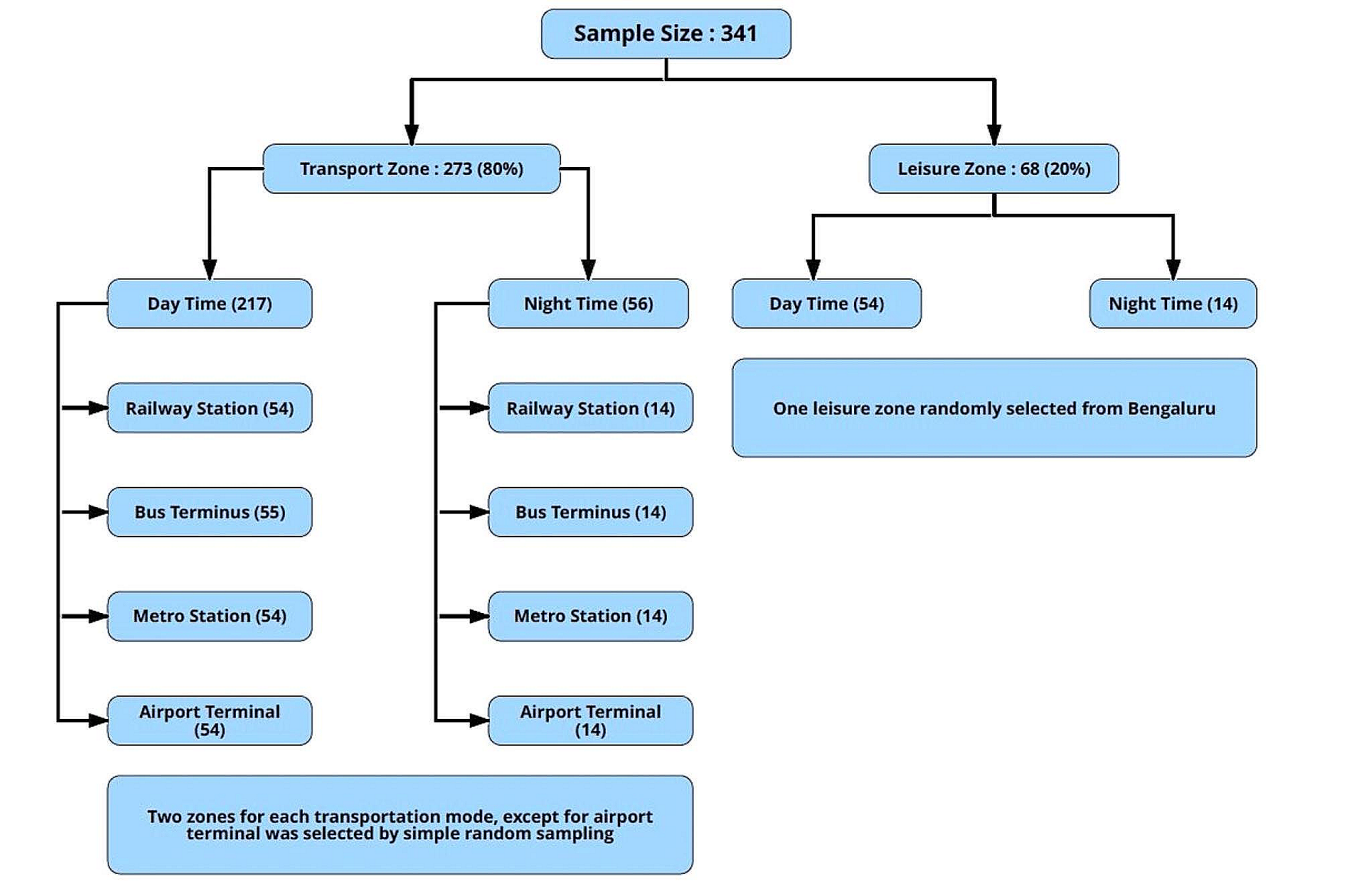
Sampling strategy

A list of transportation and leisure zones served as the sampling frame, from which two transportation zones of each type (bus, train, and metro) were selected by simple random sampling in the second stage. As driver and work environment factors differed by night and day shift, a time-period sampling was adopted to sample the study units. Within each sampled zone, 80% of the drivers were interviewed during the day (6 am to 7 pm) and 20% during the night shift (9 pm to 11 pm). An equal number of drivers were sampled from the randomly selected transportation zone. Similarly, one leisure zone was randomly sampled. Details are depicted in Fig. 1.

The following parameters were considered for sample size estimation; relative precision of 25%, confidence level of 95%, expected prevalence of adverse work environment variables among those with NCD risk factors as 50%, expected odds ratio as 1.5, and a non-response of 20%. The sample size was estimated at 340 drivers using the following formula n_a_ = [(Z^2^_α/2_ ÷ ((log^2^(1-RP))] × (1/X + 1/Y).

where, X = 1 / ρp(1-ρp) k, and Y = 1 / ρa(1-ρa), and Zα/2 is the critical value of the Normal distribution at α/2 (e.g., for a confidence level of 95%, α is 0.05 and the critical value is 1.96), RP is the relative precision (the percentage by which the lower limit for the confidence interval is less than the estimated odds ratio), ρp is the prevalence of the outcome in the presence group, ρa is the prevalence of the outcome in the absence group, and k is the ratio of presences to absences being sampled (np/na).

Each eligible ABCA driver was contacted in person by the investigator and informed verbal/written consent was obtained from the drivers after explaining the study objectives. Initially, the interviews were conducted personally, and later telephone-based video call interviews were made due to the COVID-19 pandemic in Bengaluru. Anthropometric measures, which were planned to be measured were not undertaken due to the same reason.

Study instrument: The interview was conducted using a semi-structured questionnaire to elicit information about work environment factors and selected NCD risk factors. The study instrument was developed after a literature review and expert consultations. Investigators reviewed 19 articles and subsequently shortlisted variables related to sociodemographic details, work environment, physical measurements, and NCD risk factors. Items adapted and modified from the STEPS instrument (WHO) was used for collecting information regarding selected NCD risk factors [9]. A draft study instrument was developed, and a pilot study was conducted in one randomly selected leisure zone, with a sample size of 22 ABCA drivers. The aim was to understand the feasibility of data collection using the draft study instrument. Based on the pilot study experience the questionnaire was finalised. The final study instrument comprised 6 sections and 106 items (see Table 1).

**Table 1:** Study instrument: sections and variables

Sl. No	Sections	No. of Items	Variables
1	**Sociodemographic details**	14	Place of interview, Gender, Age, Religion, Residence, Years of stay, Marital status, Living with family, Number of children, Education, Income
**Work environment**			
2	**Driver related factors**	3	History of NCD in any family member: Father/mother/Sibling/Spouse H/o Medications for NCD
3	**Driving Related factors**	19	Number of working hours, Number of days of work, Predominant working shift, if taking weekly off hours of driving per day, Seat belt usage, Mobile phone usage, Speeding, Overtaking, Crashes in the past year
4	**Vehicle-related factors**	12	Type of car, Own/rented, bought in, Serviced in, Any major repair, Insurance
5	**Welfare related factors**	6	Salary, Incentives, Medical Insurance, Accident Insurance, Vehicle Insurance
6	**NCD-related risk factors**	46	Tobacco consumption, Alcohol Consumption, Blood pressure, Height, Weight, BMI Physical activity, Diet history: Intake of fruits, vegetables

Measuring work-environment adversity (WEA): The work environment adversity was operationally defined using the ‘Work environment adversity score’ calculated from response to certain work-environment related questions answered by the drivers (See Supplementary table). Each item about assessment of the work environment was scored as 1 & 0 to calculate the work environment adversity score. A score of 0 referred to no adversity. The higher the work environment score, the higher the work environment adversity experience. The maximum score possible was 10. Based on the scores, the work environment adversity was divided into two categories for conducting logistic regression; Category 1 (Score between (0–4) and Category 2 [5–10]. These categories served as independent variables and a proxy for summarizing or quantification of work environment exposure for each driver.

Statistical analysis was done using SPSS Version 20 and STATA. Frequencies and percentages were used to summarize categorical data and mean, standard deviation was used to analyze numerical data. The prevalence of NCD risk factors was presented per 100 ABCA drivers along with 95% confidence interval estimates. Univariate analysis was conducted using Chi-square and Independent T-test to test for association between NCD risk factor (present, not present) and work environment for categorical and numerical (age, driving hours, working days) variables respectively. Independent t-test was applied to test for significant mean differences between drivers with NCD risk factors. Tests were considered significant at *p* < 0.05.

Multivariate Logistic regression analysis was conducted to quantify the strength of the association between work environment adversity categories and NCD risk factors. The Wald test was used to find out if explanatory variables in the regression model are significant. The goodness of fit was assessed using The Hosmer-Lemeshow test (HL test). Odds Ratios and 95% Confidence Intervals were provided to estimate the strength of the association. The ethical clearance was obtained from NIMHANS (National Institute of Mental Health and Neurosciences) Ethics Committee No. NIMH/DO/IEC (BS & NS DIV)2019-20.

## Results

A total of 341 eligible drivers were recruited for the study (Response rate = 99.7%). Of the 340 interviewed, 77% of drivers were aged < 40 years and 63% were from Bengaluru. Around 75% were currently married, stayed with their family, and earned a median income of approximately 40,000 per month (See supplementary table S1).

**Table 2:** Prevalence of selected NCD risk factors among cab drivers with 95% confidence intervals (*N* = 340)

	Overweight *n* (%)	Unhealthy Diet *n* (%)	Physical inactivity *n* (%)	Tobacco *n* (%)	Alcohol Use *n* (%)
**Overall**	157 (46.2) (41.0-51.5)	257 (75.6) (70.6–79.9)	265 (77.9) (73.2–82.0)	75 (22.1) (18.0-26.8)	57 (16.8) (13.2–21.1)
**Age**					
*≤* 30	58 (36.9) (29.8–44.7)	100 (38.9) (33.2–45.0)	96 (36.2) (30.7–42.2)	22 (29.3) (20.2–40.4)	12 (21.1) (12.5–33.3)
> 30	99 (63.1) (55.3–70.2)	157 (61.1) (55.0-66.8)	169 (63.8) (57.8–69.3)	53 (70.7) (59.6–79.8)	45 (78.9) (66.7–87.5)
**Education**					
Lower Primary/ High school	68 (43.3) (35.8–51.1)	103 (40.0) (34.3–46.2)	110 (41.5) (35.7–47.5)	44 (58.7) (47.4–69.1)	26 (45.7) (33.4–58.4)
Pre- University/ITI	57 (36.3) (29.2–44.1)	100 (38.9) (33.2–45.0)	107 (40.4) (34.7–46.4))	18 (24.0) (15.8–34.8)	21 (36.8) (25.5–49.8)
Degree and above	32 (20.4) (14.8–27.4)	54 (21.0) (16.5–26.4)	48 (18.1) (13.9–23.2)	13 (17.3) (10.4–27.4)	10 (17.5) (9.8–29.4)
**Marital Status**					
Currently Married	120 (76.4) (69.2–82.4)	178 (69.3) (63.4–74.6)	199 (75.1) (69.6–80.0)	57 (76.0) (65.2–84.2)	45 (78.9) (66.7–87.5)
Never Married	37 (23.6) (17.6–30.8)	79 (30.7) (25.4–36.6)	66 (24.9) (20.1–30.5)	18 (24.0) (15.8–34.8)	12 (21.1) (12.5–33.3)
**Staying with Family**					
Yes, everyday	96 (61.1) (53.3–68.4)	154 (59.9) (53.8–65.7)	164 (61.9) (55.9–67.5)	51 (68.0) (56.8–77.5)	37 (64.9) (51.9–76.0)
Yes, only during non-working days	59 (37.6) (30.4–45.4)	95 (37.0) (31.3–43.0)	95 (35.8) (30.3–41.8)	22 (29.3) (20.2–40.4)	19 (33.3) (22.5–46.3)
No	2 (1.3) (0.4–4.5)	8 (3.1) (1.6-6.0)	6 (2.3) (1.0-4.9)	2 (2.7) (0.7–9.2)	1 (1.8) (0.3–9.3)

### Prevalence of NCD risk factors among cab drivers

Nearly 97% of drivers reported having one or more NCD risk factors. We observed that 22.1% of the drivers were tobacco users and 16.8% were alcohol users. Most (78%) of the participants were physically inactive (See Table 2). The mean Weight and BMI were found to be 75.3 kg and 27.7 respectively.

**Table 3:** Work duration of driver and NCD risk factors (*N* = 340)

Work-Related Variables	Overall	Overweight	Physical inactivity	Unhealthy Diet	Tobacco Use	Alcohol Use
	*N* = 341N = 340	*N* = 156	*N* = 265	*N* = 257	*N* = 75	*N* = 57
	Mean ± SD	Mean ± SD	Mean ± SD	Mean ± SD	Mean ± SD	Mean ± SD
**Working Hours (per day)**	8.7 ± 1.5	8.6 ± 1.4	8.7 ± 1.5	**8.8 ± 1.6** ^*^	**9.1 ± 2.2** ^*^	**7.9 ± 1.4** ^*^
**Working Hours (per Night)**	3.9 ± 2	4.1 ± 1.9	4.0 ± 2.0	4.3 ± 2.0*	5.0 ± 2.3*	4.1 ± 1.8
**Average Km/day**	89.3 ± 19.8	89.9 ± 17.5	89.0 ± 18.1	90.4 ± 20.1	89.3 ± 17.7	84.2 ± 25.2
**Average Km/night**	30.4 ± 15.7	**33.7 ± 15.3** ^*^	40.0 ± 15.9	31.6 ± 16.1*	26.5 ± 17.8*	37.7 ± 16.4*
**Days per week**	5.9 ± 0.4	**6.0 ± 0.3** ^*^	**5.9 ± 0.4** ^*^	5.8 ± 0.5*	5.9 ± 0.4	5.9 ± 0.3
**Trips per day**	8.9 ± 2	9.0 ± 1.7	8.9 ± 1.8	9.0 ± 2.0	9.0 ± 2.6	8.4 ± 2.5
**Trips per Night**	3 ± 1.6	**3.4 ± 1.5** ^*^	3.4 ± 1.5	3.2 ± 1.6*	2.7 ± 1.8*	3.8 ± 1.6*

Note: * means the value is statistically significant with *p* < 0.05, Independent T test

**Table 4:** Association between work environment factors and selected NCD risk factors with 95% confidence intervals (*N* = 340)

Variables	*N*	Physical inactivity		Overweight		Unhealthy diet		Tobacco Use		Alcohol Use	
		OR	AOR	OR	AOR	OR	AOR	OR	AOR	OR	AOR
Staying away from family	120	1.56 (0.89–2.73)	1.38 (0.75–2.56)	1.30 (0.84–2.03)	1.16 (0.72–1.84)	2.22 (1.25–3.92)	2.85* (1.52–5.34)	0.81 (0.47–1.41)	0.90 (0.46–1.75)	0.97 (0.54–1.77)	0.80 (0.43–1.50)
Working 5 + days a week	277	3.40 (1.86–6.19)	3.46* (1.70–7.06)	3.72 (1.93–7.19)	4.07* (1.98–8.33)	0.24 (0.19–0.63)	0.30* (0.11–0.86)	1.00 (0.51–1.97)	5.52 (2.10-14.48)	1.96 (0.80–4.80)	1.47 (0.53–4.05)
Working 7 + hours a day	227	2.10 (1.24–3.56)	1.44 (0.72–2.85)	1.42 (0.89–2.24)	2.16* (1.16-4.00)	5.09 (3.00-8.610	4.84* (2.32–10.08)	2.44 (1.29–4.59)	9.33* (3.80-22.97)	0.85 (0.47–1.55)	0.36 (0.14–0.95)
Working predominantly at night	144	4.03 (2.18–7.45)	2.49* (1.16–5.35)	1.25 (0.81–1.92)	0.75 (0.42–1.35)	2.47 (1.44–4.24)	1.08 (0.50–2.32)	0.58 (0.34–0.99)	0.31 (0.15–0.64)	2.19 (1.23–3.91)	4.64* (1.81–11.90)
Medical Insurance absent	64	2.99 (1.67–5.34)	2.53* (1.29–4.98)	0.89 (0.52–1.53)	0.77 (0.42–1.40)	2.08 (1.17–3.17)	1.41 (0.72–2.78)	0.94 (0.49–1.81)	0.31 (0.14–0.72)	0.62 (0.32–1.19)	0.46* (0.22–0.98)
Road crashes in the past	49	0.42 (0.22–0.80)	0.70 (0.30–1.63)	0.57 (0.31–1.08)	0.70 (0.32–1.48)	1.78 (0.80–3.97)	1.45 (0.56–3.70)	2.96 (1.56–5.62)	0.98 (0.39–2.44)	0.29 (0.09–0.96)	0.38 (0.11–1.38)
Seized by Traffic police	99	0.99 (0.56–1.73)	1.16 (0.62–2.18)	0.60 (0.37–0.97)	0.65 (0.39–1.07)	1.29 (0.73–2.26)	0.92 (0.49–1.72)	2.04 (1.19–3.49)	2.26* (1.20–4.25)	1.15 (0.62–2.13)	1.23 (0.63–2.43)
Vehicle score	70	0.77 (0.42–1.42)	1.18 (0.52–2.68)	0.73 (0.43–1.24)	1.29 (0.67–2.50)	1.54 (0.79–2.97)	1.42 (0.62–3.23)	6.2 (3.52–11.19)	17.08* (7.01–41.60)	0.91 (0.44–1.86)	1.75 (0.74–4.15)

Note: * means the value is statistically significant with *p* < 0.05, Logistic regression analysis

### Work environment adversity and NCD risk factors

Table 3 provides an overview of the work duration of the cab drivers and NCD risk factors among them. Drivers were found to be working for 8.7 hours per day on an average and close to 4 hours during night, six days a week. Drivers completed an average of 9 trips during day shift and 3 trips during night shift. Around 58% of ABCA drivers have a work adversity score of 5 or more suggesting higher adverse working environment conditions (see supplementary table S3). A total of 9 work-related variables were considered and tested for association with NCD risk factors among the ABCA drivers. Staying away from family, and long working hours with predominant night shifts were significantly associated with an unhealthy diet. Working more than 6 days per week was also significantly associated with drivers being overweight. A significant association was found between substance use and night shifts as well as road crashes in the past. Similarly, adverse working conditions like working 7 + hours/day more than 6 days a week, and predominant night shifts were linked to NCD risk factors, and physical inactivity (See supplementary table S4).

Table 4 notes that drivers working more hours (> 7 h) were almost at a 5 times higher risk of consuming an unhealthy diet compared to those who worked less than 7 h a day. Working more than 5 days per week put the drivers at 3.46 times higher risk of being physically inactive and at four times (AOR = 4.07) higher risk of becoming overweight compared to those who worked less than 5 days per week. The odds of using tobacco were 9.3 times higher among drivers working 7 + hours a day than those who drove less than 7 h a day. Drivers who did night shifts were found to be four times (AOR = 4.64) at a greater risk of consuming alcohol. Work adversity score between 5 and 10 was associated with higher odds of Physical Inactivity (OR = 3.1), Unhealthy diets (1.62), and Tobacco Use (OR = 3.06) (See Table 5).

**Table 5:** Multivariable logistic Regression showing an association between NCD risk factors and Work Environment Adversity Score Categories

NCD Risk Factors	Total	Exp(B)	95% C.I. for Exp(B)	
	*N* (%)		Lower	Upper
Tobacco use	75(22.1)	3.069*	1.595	5.905
Alcohol use	57(16.8)	1.277	0.653	2.500
Overweight	157(46.2)	1.018	0.634	1.636
Physical Inactivity	265(77.9)	3.170*	1.786	5.626
Unhealthy diet	257(75.6)	1.627*	0.957	2.767

Note: * means the value is statistically significant with *p* < 0.05, Logistic regression analysis

## Discussion

This study is one of the few studies exploring associations between work environment adversity and NCD risk factors among ABCA drivers in India. The data collection started in March 2020 and was discontinued due to COVID-19-related cab service disruptions and was later resumed in the 3rd week of July 2020. The duration of sample collection was limited as the pandemic had drastically reduced the number of cabs available and was permitted only to cater to the needs of primary health care services and emergencies at different times. Despite the pandemic, we were able to complete data collection with a high response rate, which is the strength of the study. Owing to the COVID-19 appropriate behavior mandates, we deferred from measuring blood pressure and anthropometric measurements.

Our sample included drivers from both transportation and leisure zones of the city, working during day and night. All the respondents were males since we were unable to trace any female taxi drivers. Though the average reported income of the drivers was 40,000/-, nearly similar to the average per capita income of Indian citizens for the year 2020 (USD 6728, Rs 452,376 per year i.e. 37,698 per month) [10], most drivers are expected to have recurrent expenditures related to loan repayment, commercial taxes, fuel, and maintenance of vehicles. Hence their affordability for health security, insurance, and compliance with health promotion interventions is compromised. Despite this, it can be presumed that there is expendable income to indulge in tobacco and alcohol use, due to the high prevalence observed.

Karnataka State Youth Policy 2012 defines Youth as “young people in the age group of 16–30.” In our study, 30.3% of the drivers belonged to this age category compared to a study done among taxi drivers by Rathi et al., where 17.4% of the drivers were aged less than 30 years [11]. With one-third of ABCA drivers being youth, there is scope for implementing a youth-focused NCD prevention program for cab drivers or referring them to ongoing youth health promotion programs like Yuva Spandana and Life Skills in Bengaluru [12].

We observed that 36.8% of the drivers were not Bengaluru residents and hence were staying away from their families and living and working from the car. This unique attribute makes them vulnerable to regularly consuming unhealthy diets, indulging in substance use, and physical inactivity. These drivers are likely to be deprived of the care and personal psychosocial support mechanisms expected from their families.

Regular consumption of high-salt foodstuffs, fried food items, and carbonated beverages was used to define unhealthy dietary practices in this study. According to our study, around 75% of cab drivers reported unhealthy diet patterns, in contrast to the literature, which indicates the prevalence of junk food consumption to be around 18.66% [11]. Differences exist due to different operational definitions of unhealthy diets. A Study conducted in South Africa among taxi drivers revealed that regular consumption of fried snacks increased the risk of developing metabolic syndrome by 3.7 times[13].

Inadequate physical activity is a well-established risk factor for premature mortality due to NCDs such as coronary heart diseases, stroke contributing to 8% of non-communicable diseases and deaths globally [14]. Drivers who work long shifts and long work hours find it challenging to engage in physical activity. Extended periods of sitting have been linked to increased risk of diabetes, obesity cardiovascular disease, and all-cause mortality. Physical inactivity coupled with consumption of energy dense food, high in sugar and fat often lead to higher BMI, resulting in higher incidence of hypertension and blood glucose [15]. In short, this group is a ticking time bomb for cardiovascular problems in the future, with over 78% of the cab drivers reporting to be physically inactive, 46% being overweight and 75% consuming unhealthy diet.

Tobacco is used by 1.33 billion people globally, with 28.6% of the population in India using tobacco in some form (GATS-2), and 28.2% in Karnataka (GATS-2) [16] According to a survey, Bengaluru cab drivers consumed tobacco at a very high rate of 70.9%, which was higher than the national average of 42.4% for all men. Smokeless tobacco products such as gutka, khaini, and zarda, were consumed by almost 55% of Bengaluru’s taxi drivers [17]. Another study by Gany et al. found that around 30% of the drivers consumed tobacco in some form [18]. However, in our study, we observed that only 22.1% of the cab drivers consumed tobacco, which is much lesser compared to both the studies. Underreporting of substance use owing to social desirability could have been the reason for the low figures in our study.

Alarmingly, among drivers who reported alcohol use (16.8%), more than 90% were identified to be dependent users and are more vulnerable to the onset and progression of NCDs. Such high levels of dependent use warrant regular and random alcohol breath testing strategies for drivers as they pose a risk not only to themselves but also to other vehicle users, pedestrians, and the general public. Functional collaboration with the district mental health program and ABCA operators needs to be strengthened to provide brief interventions and de-addiction services.

We observed a higher risk for NCD risk factors among drivers working > 5 days a week, 7 + hours a day, staying away from family, and working night shifts. This is in line with a web of causation of NCDs wherein higher stressful work and longer working hours are associated with NCDs. Hence, results indicate a need for better strategies to monitor and address the issues of working hours, workdays, and family support.

With the high prevalence of NCDs, and NCD risk factors among an estimated 1.6 lakh registered cab drivers in Bengaluru, the proportion requiring NCD prevention and care would be significant. If we consider the entire country, the figures represent a sizeable group. It certainly calls for a concerted effort to address the issue of NCDs among drivers. The study has limitations in that it is not a representative sample of all cab drivers of Bengaluru. The work environment adversity score has scope for validity assessment across a bigger sample size.

A report released by IFAT elaborated the stressful work environment of ABCA drivers in India. 89.8% of the respondents claimed they get less than 6 h of sleep, 39.8% of the respondents spent close to 20 h in work and the lack of health insurance caused them to ignore their ailments. A significant number of respondents felt that the isolated nature of their work has affected their consumption habits of alcohol and tobacco which has incrementally increased over time [19].

In India, the existing occupational health programs do not cater to ABCA drivers, who are employed on a contract basis and are not subject to any specific laws or regulations. There are no explicitly mentioned frameworks or strategies in existing health programs to address ABCA drivers nor there is NCD risk factor surveillance in this population.

The study highlights the association between work environment adversity and NCD risk factors and indicates a dire need for Basic Occupational Health Services and social security legislation/provisions for this working population. It implies a need for government, company and individual related interventions to reduce NCD risk among ABCA drivers. The intervention will focus on setting up systems for measuring and monitoring the work environment adversity of ABCA drivers, regular screening for NCDs by periodical health examination, providing specific health promotion interventions, and establishing linkages with ongoing health programmes for management of NCDs and mental disorders. We also recommend conduction of a larger representative sample survey in more cities across the country to arrive at more precise estimates. Periodical screening for NCDs and risk factors is recommended on an annual basis. As a short-term measure, there is a need for outreach strategies in national programs and NCDs & mental health (NP-NCD and NMHP) to ensure access to health promotion, screening, and treatment services for ABCA drivers in Bengaluru. Stakeholders’ meetings (ABCA companies, cab drivers, unions, health department, and labor department) are recommended to debate and discuss strategies to reduce NCDs in ABCA drivers. An innovative way of monitoring working hours and driving time without affecting the revenues of drivers, using a technology approach is recommended.

## Conclusion

The study concluded that NCD risk factors like physical inactivity, unhealthy diets, overweight, tobacco use, and alcohol use are commonly prevalent among ABCA drivers in Bengaluru. Working more than 5 days a week, more than 7 + hours a day, staying away from family, and working night shifts are closely associated with higher risk for NCD risk factors among ABCA drivers. The risk for NCD risk factors also is higher among drivers with a work adversity score of more than five.

In this regard, the findings of the study justify the need for NCD prevention services including Basic Occupational Health Services for ABCA drivers in Bengaluru city. Given the findings, there is a need for larger representative studies across all Indian metropolitan cities followed by developing a need-based and customized setting-based intervention package for reducing NCDs and substance use among ABCA drivers in India.

### Electronic supplementary material

Below is the link to the electronic supplementary material.

Supplementary Material 1

## Data Availability

All data generated or analysed during this study are included in this published article [and its supplementary information files]. Any further information/clarification required is available from the corresponding author on reasonable request.
