# Supplementary material for: Work environment adversity and non-communicable Disease risk among drivers working for application-based-cab-aggregators in an Indian metropolis: Assessment of work environment adversity and its association with ncd risk factors among application based cab aggregators in Bengaluru, India: a cross sectional study
Source: BMC Public Health. 2024 Jun 14;24:1592. doi: 10.1186/s12889-024-18728-y (PMC11177478; doi:10.1186/s12889-024-18728-y)
Supplement: Supplementary file 1 — Supplementary Material 1 [file 12889_2024_18728_MOESM1_ESM.docx]

**Supplementary tables**

**Table S1: Socio-demographic details of study subjects**

| **Sociodemographic characteristics (n=340)** | **N (%)** |
| --- | --- |
| **Age (in years)** | |
| < 30 | 103(30.3) |
| >30 years | 237(69.7) |
| Age (in years) (Mean ± SD) | 34.8±7.2 |
| **Permanent Residence** | |
| Bengaluru | 215(63.2) |
| Others | 125(36.8) |
| **Education** | |
| Not Literate | 0(0.0) |
| Lower Primary School | 10(2.9) |
| Up to High school | 137(40.3) |
| Pre- University/ITI | 133(39.1) |
| Degree and above | 60(17.6) |
| **Marital Status** | |
| Currently married | 258(75.9) |
| Never married | 82(24.1) |
| Separated/ divorced/ widowed | 0(0.0) |
| **Staying with family (n=258)** | |
| Yes, everyday | 187(72.5) |
| Yes, only during non-working days | 66(25.6) |
| No | 5(1.9) |
| **Number of Living Children (n=258)** | |
| 0 | 28(10.9) |
| 1-2 | 199(77.1) |
| 3 and above | 31(12.0) |
| **Income per month (in rupees)** | |
| Mean ± SD | 39,603 ± 11,290 |
| Median | 40,000 |

**Table S2: Work Environment Score for ABCA drivers**

| **Sl. No** | **Sections** | **Criteria** | **Score** |
| --- | --- | --- | --- |
| A. Driver Related factors | | | |
| 1. | Driving hours | 6+hours/day | 1 |
|  |  | <6hours/day | 0 |
| 2. | working days per week | 5+days/week | 1 |
|  |  | <5days/week | 0 |
| 3. | Predominant working shift | Night | 1 |
|  |  | Day | 0 |
| 4. | Road crashes in past one year | Yes | 1 |
|  |  | No | 0 |
| 5. | Staying away from family | Yes | 1 |
|  |  | No | 0 |
| 6. | Fined by traffic police during past one year | Yes | 1 |
|  |  | No | 0 |
| B. Vehicle Related Factors | | | |
| 7. | Vehicle ownership | Rented | 1 |
|  |  | Own | 0 |
| C. Welfare Related Factors | | | |
| 8. | Salary credited on time | No | 1 |
|  |  | Yes | 0 |
| 9. | Medical Insurance | No | 1 |
|  |  | Yes | 0 |
| 10. | Incentives provided | No | 1 |
|  |  | Yes | 0 |

**Table S3: Frequency of Work adversity scores among cab drivers**

| **WORK ADVERSITY SCORE** | **FREQUENCY** | **PERCENT** | **CUMULATIVE PERCENT** |
| --- | --- | --- | --- |
| **0** | 4 | 1.2 | 1.2 |
| **2** | 16 | 4.7 | 5.9 |
| **3** | 57 | 16.8 | 22.6 |
| **4** | 65 | 19.1 | 41.8 |
| **5** | 100 | 29.4 | 71.2 |
| **6** | 75 | 22.1 | 93.2 |
| **7** | 21 | 6.2 | 99.4 |
| **8** | 2 | 0.6 | 100.0 |
| **Total** | **340** | **100.0** |  |

**Table S4: Association between NCD risk factors and adverse work environment**

| **Variables** | **Overweight** | **Chi square test** | **Physical Inactivity** | **Chi square test** | **Unhealthy Diet** | **Chi square test** | **Tobacco Use** | **Chi square test** | **Alcohol Use** | **Chi square**  **test** |
| --- | --- | --- | --- | --- | --- | --- | --- | --- | --- | --- |
|  | Reported  N=157 |  | Reported  N=265 |  | Reported  N=257 |  | Reported  N=75 |  | Reported  N=57 |  |
| **Driver Related Factors** |  |  |  |  |  |  |  |  |  |  |
| Staying away from family | 61(38.9) | 1.12 | 101(38.1) | 2.60 | 103(40.1) | **8.05*** | 22(30.6) | 1.13 | 20(35.1) | 0.02 |
| Working 7+ hours/day | 113(72.0) | 2.18 | 190(71.7) | **7.79^*^** | 198(77.0) | **40.04*** | 58(80.6) | **6.67^*^** | 37(64.9) | 0.29 |
| Working 6+days/week | 144(91.7) | **16.74*** | 231(87.2) | **17.13*** | 203(79.0) | **9.83*** | 59(81.9) | 0.03 | 51(89.5) | 2.23 |
| Predominantly working night shift | 73(46.5) | 1.05 | 133(50.2) | **21.67*** | 125(48.6) | **11.18*** | 22(30.6) | **6.26*** | 34(59.6) | **7.24^*^** |
| Road crashes in past 1 year | 17(10.8) | 3.04 | 31(11.7) | **7.17*** | 41(16.0) | 2.03 | 20(27.8) | **13.23*** | 3(5.3) | **4.65*** |
| Seized by traffic police | 37(23.6) | **4.36*** | 77(29.1) | 0.00 | 78(30.4) | 0.78 | 31(43.1) | **8.60*** | 18(31.6) | 0.20 |
| **Vehicle Related factors** |  |  |  |  |  |  |  |  |  |  |
| Driving Rented car | 28(17.8) | 1.35 | 52(19.6) | 0.69 | 57(22.2) | 1.63 | 36(50.0) | 48.33 | 11(19.3) | 0.07 |
| **Welfare Related Factors** |  |  |  |  |  |  |  |  |  |  |
| Medical Insurance Absent | 125(79.6) | 0.18 | 225(84.9) | **14.31*** | 215(83.7) | 6.34 | 58(80.6) | 0.00 | 42(73.7) | 2.09 |
| No Incentives Given | 139(88.5) | 0.00 | 247(93.2) | 0.02 | 239(93.0) | 0.01 | 61(84.7) | **18.16*** | 57(100) | 0.05 |

Note: * means the value is statistically significant with p<0.05, chi square/ Fischer’s exact test
